# Molecular Characterization of New Haplotype of Genus *Sarcocystis* in Seabirds from Magdalena Island, Southern Chile

**DOI:** 10.3390/ani11020245

**Published:** 2021-01-20

**Authors:** Igor C. L. Acosta, Solange M. Gennari, Horwald A. B. Llano, Sebastián Muñoz-Leal, Rodrigo M. Soares

**Affiliations:** 1 Department of Preventive Veterinary Medicine and Animal Health, Faculty of Veterinary Medicine, University of São Paulo, Avenida Prof. Orlando Marques de Paiva, 87, São Paulo CEP 05508-270, Brazil; sgennari@usp.br (S.M.G.); horwald@hotmail.com (H.A.B.L.); sebamunoz@udec.cl (S.M.-L.); rosoares@usp.br (R.M.S.); 2 PhD Program in Medicine, Animal Welfare and Public Health, Faculty of Veterinary Medicine, University Santo Amaro, Rua Prof. Enéas de Siqueira Neto, 340, São Paulo CEP 04829-300, Brazil; 3 Department of Pathology and Preventive Medicine, Faculty of Veterinary Sciences, University of Concepción, Avenida Vicente Mendez, 595, Chillán CEP 3780000, Chile

**Keywords:** wild birds, coccidian, molecular, apicomplexa, marine

## Abstract

**Simple Summary:**

Sarcocystidae is a family of apicomplexan protozoa highly prevalent in vertebrates. The definitive hosts of sarcocystids eliminate oocysts or sporocysts that infect intermediate hosts. After infection, mature tissue cysts (sarcocysts) develop in intermediate hosts, mostly in muscle and neurological tissues. *Sarcocysts* are infectious for definitive hosts, which acquire them through carnivorous or scavenging habits. Intermediate hosts and definitive hosts are the natural hosts of sarcocystids in which infections are usually mildly or not symptomatic. In 2017, muscular and neurological tissues of 22 birds from Magdalena Islands, southern coast of Chile, were screened for the presence of DNA of sarcocystids. DNA of organisms of the genus *Sarcocystis* was identified in two Chilean skuas (*Stercorarius chilensis*). The genetic makeup of the parasite detected in skuas was unprecedented and probably represent a new species in the genus. It is well known that *Sarcocystis* may cause severe infections in aberrant hosts, which are susceptible animals that do not behave as natural hosts for the parasite and have low resistance to the infection, thus more studies are needed to characterize this parasitosis in skuas and other hosts to understand the epidemiology of the infection and its impact on the health of marine fauna.

**Abstract:**

Evidence of sarcocystid infection was investigated in samples of 16 penguins (*Spheniscus. magellanicus),* four Dominican gulls (*Larus dominicanus*) and two Chilean skuas (*Stercorarius chilensis*) found in Madalenas Islands, Chile, in 2017. Samples of skeletal muscle, cardiac muscle and brain from all birds were screened by a pan-sarcocystid nested-PCR targeting a short fragment of the gene encoding the small ribosomal unit (nPCR-18Sa). The only two positive samples by nPCR-18Sa, both from skuas, were tested by a nested-PCR directed to the internal transcribed spacer 1 (nPCR-ITS1), also a pan-sarcocystidae nested-PCR, and to a nested-PCR directed to the B1 gene (nPCR-B1), for the exclusive detection of *Toxoplasma gondii*. The two nPCR-18Sa-positive samples were nPCR-ITS1-positive and nPCR-B1-negative. The nPCR-ITS1 nucleotide sequences from the two skuas, which were identical to each other, were revealed closely related to homologous sequences of *Sarcocystis halieti*, species found in seabirds of northern hemisphere. Larger fragments of genes encoding 18S and partial sequences of genes coding for cytochrome oxidase subunit 1 were also analyzed, corroborating ITS1 data. The haplotypes found in the skuas are unprecedent and closely related to species that use birds as the definitive host. Further studies need to be carried out to detect, identify and isolate this parasite to understand the epidemiology of the infection and its impact on the health of marine fauna.

## 1. Introduction

The phylum Apicomplexa is composed of obligate intracellular parasites that are characterized by having a specialized structure called the apical complex, which is used to invade vertebrate host cells [1] Within this phylum, the Sarcocystidae family comprises more than 196 species of coccidia that form cysts in tissues of intermediate hosts. Although taxonomic controversies still exist, this family has been divided into three subfamilies: Sarcocystinae, represented by the genera *Frenkelia* and *Sarcocystis*; Cystoisosporinae, containing the genus *Cystoisospora*; and Toxoplasmatinae, a subfamily with a few species grouped in the genera *Besnoitia*, *Hammondia*, *Neospora* and *Toxoplasma* [2,3,4,5].

*Toxoplasma gondii* is a coccidian parasite with worldwide distribution. It infects virtually all warm-blooded animals, including humans, but only cats (domestic and wild) act as definitive hosts. Toxoplasmosis has been reported in many avian species; however, little information is available in relation to populations of *Spheniscus magellanicus*, *Stercorarius chilensis* and *Larus dominicanus* [6]. Recently, *T. gondii* antibodies were detected in 57 (43.18%) out of 132 serum samples collected from free-living Magellanic penguins (*Spheniscus magellanicus)* on Magdalena Island, Chile, with titers that ranged from 20 to 320 [7].

The genus *Sarcocystis* has an obligate two-host life cycle. Asexual stages develop in intermediate hosts, usually omnivores, through forming cysts in the musculature and central nervous system. Infection of intermediate hosts occurs through their ingestion of food or water contaminated with sporocysts. Sexual stages only develop in the definitive host, which is typically a carnivore or an omnivore, and infection in this case occurs through ingestion of meat contaminated with cysts [8]. Sarcocystids of the genus *Sarcocystis* may cause severe infections in aberrant hosts, which are susceptible animals that do not behave as natural hosts of the parasite and have low resistance to the infection. Thus, *Sarcocystis* potentially pose risk to human and animal health, depending on the susceptible host behaving as aberrant host or not [8].

A few studies have documented the presence of *Sarcocystis* spp. in wild animals in Chile. Presence of cysts of this parasite has been confirmed in muscle tissues of pudu deer (*Pudu puda*), guanacos (*Lama guanicoe*) and sea lions (*Otaria byronia)* [9,10,11]. However, *Sarcocystis* has not yet been described in Chilean wild birds.

The Chilean skua *Stercorarius chilensis* is a large predatory seabird that inhabits shore ecosystems along the southern cone of South America from central Peru to northern Argentina, with occasional occurrence on the coasts of Ecuador, Brazil, Uruguay and Antarctica [12]. Skuas belong to the order Charadriiformes and are considered to be opportunist feeders, preying on a wide diversity of animals such as small seabirds, fish, scraps and carrion [13,14]. Populations of skuas may be small, but they do not approach the thresholds for vulnerable classification following a population-size criterion (<10,000 mature individuals) [15].

Considering other coastal birds’ species, the Kelp Gull (*Larus dominicanus*) is an opportunistic feeder like numerous Laridae and consumes a wide variety of fishes, invertebrates and fisheries waste [16,17]. A high diversity in the use of habitat types has been recorded throughout its distributional range in the Southern Hemisphere, including Argentina, Brazil, Chile, Peru and Uruguay, and the breeding population has been estimated at least 160,000 pairs [17]. In contrast, the Magellanic penguin (*S. magellanicus*) has approximately 1.1 to 1.6 million breeding pairs that nest along the eastern and western coasts of South America, in Argentina Chile and the Malvinas/Falkland Islands [18]. *Spheniscus magellanicus* has a primarily piscivorous diet with the presence of some cephalopods and crustaceans [19].

To date, more than 25 species of *Sarcocystis* have been found to use birds as intermediate hosts [8,20]. *Sarcocystis falcatula*, *Sarcocystis calchasi* and the recently described unnamed species *Sarcocystis* sp. Chicken-2016-DF-BR, which can possibly be interpreted as *Sarcocystis wenzeli* [21] are species that may be pathogenic for intermediate hosts [22,23,24].

Focusing on *T. gondi* and the genus *Sarcocystis*, the aim of this study was to screen for the evidence of new species or species genotypes of Sarcocystidae in seabird carcasses from southern Chile, a region with scarce data on the occurrence of this group of parasites. Molecular evidence of a unique haplotype of genus *Sarcocystis* was found in two Chilean skuas (*S. chilensis*).

## 2. Materials and Methods

### 2.1. Ethical Considerations

Sample collections on Magdalena Island were conducted under license no. 039/2016 issued by the National Forestry Corporation (Corporación Nacional Forestal; CONAF), and permit no. 2799 issued by the National Fisheries Service (Servicio Nacional de Pesca; SERNAPESCA), Chile. This study was approved by the Ethics Committee on Animal Use (CEUA-no. 9701041113) of the School of Veterinary Medicine, University of São Paulo (FMVZ-USP).

### 2.2. Collection of Samples

In January 2017, fragments from the pectoral muscle, heart and brain, comprising approximately 5–10 g each, were collected from fresh seabird carcasses on Magdalena Island. This island is located in the Strait of Magellan, near the city of Punta Arenas, in southern Chile (52°55′10.0″ S; 70°34′37.7″ W), and constitutes a natural reserve named “Monumento Natural Los Pinguinos”. Necropsies were performed in situ and samples were stored in sterile microtubes at −20 °C until the time of analysis. Samples were collected from 22 birds: 16 penguins (*S. magellanicus*), four Dominican gulls (*L. dominicanus*) and two Chilean skuas (*S. chilensis*), totaling 66 samples (22 from pectoral muscles, 22 from hearts and 22 from brains).

### 2.3. Molecular Identification

Tissue samples of 25–50 mg were subjected to DNA extraction using the DNeasy Blood and Tissue kit (Qiagen, Hilden, Germany) following the manufacturer’s recommendations, with the exception of final elution of the product into 50 µL of elution buffer from Qiagen Kit. As internal control for the evaluation of the successfulness of the DNA extraction, DNA samples were tested by conventional PCR targeting mitochondrial hypervariable region in the penguins derived samples and by PCR directed to mitochondrial 16S rRNA gene in the tissues from the other seabirds [25,26,27].

Initial screening targeting the Sarcocystidae family was performed using a pan-sarcocystid nested PCR based on primers [28] directed to a short fragment of 18S rDNA gene (nPCR-18Sa). The nPCR-18Sa positive samples were further investigated for the presence of DNA of *T. gondii* to amplify partial fragments of gene B1 (nPCR-B1) using the primers described by [29]. The nPCR-18Sa positive samples were also tested by a second pan-sarcocystid nested PCR directed to internal transcribed spacer 1 (nPCR-ITS1) [30,31] The nPCR-ITS1 were used in order to obtain genetic sequences capable of differentiating the species of the Sarcocystid screened with nPCR-18Sa. The nPCR-ITS1 positive samples were further tested with a third pan-sarcocystid nested PCR, now targeting a larger fragment of 18S rDNA gene (nPCR-18Sb) using primers described by [32], as well with a *Sarcocystis* specific nested PCR [30] directed to cytochrome oxidase subunit I (nPCR-CO1). The primers are depicted in Table 1.

The first round of nPCR-18Sa were performed with 3.0 µL of extracted DNA, 1.8 µL of 10× PCR Buffer (KCL 50 mM; Tris HCl 10 mM; pH 9.0) (Life Technologies Corporation, Carlsbad, CA 92008 USA), 0.7 µL of MgCL_2_ (50 mM), 1.4 µL of mixed dNTPs (10 mM), 0.1 µL of each primer (25 µM), 0.14 µL of Platinum^TM^ Taq DNA Polymerase (5 U/µL) (Life Technologies Corporation, Carlsbad, CA 92008 USA) and ultrapure autoclaved water to a volume of 18 µL per reaction. The PCR thermal cycling consisted of an initial incubation at 94 °C for 30 sec, followed by 30 cycles (94 °C for 25 sec, 55 °C for 1 min, 72 °C for 1.5 min) and a final extension at 72 °C for 10 min. For the second rounds: 1 µL of template derived from the first reactions, 2.5 µL of 10× PCR Buffer (KCL 50 mM; Tris HCl 10 mM; pH 9.0) (Life Technologies Corporation, Carlsbad, CA 92008 USA), 2.5 µL of MgCL_2_ (50 mM), 4.0 µL of mixed dNTPs (10 mM), 1.25 µL of each primer (10µM), 0.15 µL of Platinum^TM^ Taq DNA Polymerase (5 U/µL) (Life Technologies Corporation, Carlsbad, CA 92008 USA)and ultrapure autoclaved water to a volume of 25 µL per reaction. The PCR thermal cycling consisted of an initial incubation at 94 °C for 4 min, followed by 30 cycles (94 °C for 30 s, 55 °C for 1 min, 72 °C for 2.0 min) and a final extension at 72 °C for 10 min. For the second rounds the same quantities of the reagent mixture with primers at 50 μM, using 2 μL of the product of the PCR diluted in ultra-pure water (1:2). The nPCR thermal cycling consisted of an initial incubation at 94 °C for 4 min, followed by 35 cycles (94 °C for 30 s, 55 °C for 1 min, 72 °C for 2.0 min) and a final extension at 72 °C for 10 min.

The first round of nPCR-B1 were performed with 1.0 µL of extracted DNA, 2.5 µL of 10× PCR Buffer (KCL 50 mM; Tris HCl 10 mM; pH 9.0), (Life Technologies Corporation, Carlsbad, CA 92008 USA), 0.75 µL of MgCL_2_ (50 mM), 4.0 µL of mixed dNTPs (10 mM), 1.25 µL of each primer (10 µM), 0.15 µL of Platinum^TM^ Taq DNA Polymerase (5 U/µL) (Life Technologies Corporation, Carlsbad, CA 92008 USA)and ultrapure autoclaved water to a volume of 25 µL per reaction. The PCR thermal cycling consisted of an initial incubation at 94 °C for 3 min, followed by 25 cycles (94 °C for 25 s, 55 °C for 1 min, 72 °C for 1.5 min) and a final extension at 72 °C for 10 min. For the second rounds: 1 µL of template derived from the first reactions, 2.5 µL of 10× PCR Buffer (KCL 50 mM; Tris HCl 10 mM; pH 9.0) (Life Technologies Corporation, Carlsbad, CA 92008 USA), 2.5 µL of MgCL_2_ (50 mM), 4.0 µL of mixed dNTPs (10 mM), 1.25 µL of each primer (10 µM), 0.15 µL of Platinum^TM^ Taq DNA Polymerase (Life Technologies Corporation, Carlsbad, CA 92008 USA) and ultrapure autoclaved water to a volume of 25 µL per reaction. The nPCR thermal cycling consisted of an initial incubation at 94 °C for 3 min, followed by 35 cycles (94 °C for 25 s, 55 °C for 1 min, 72 °C for 1.5 min) and a final extension at 72 °C for 10 min.

The first round of nPCR-18Sb, nPCR-ITS1 and nPCR-CO1 were performed with 4 µL of extracted DNA, 2.5 µL of 10× PCR Buffer (KCL 50 mM; Tris HCl 10 mM; pH 9.0) (Life Technologies Corporation, Carlsbad, CA 92008 USA), 1.0 µL of MgCL_2_ (50 mM), 0.5 µL of mixed dNTPs (10 mM), 1.0 µL of each primer (10 µM), 0.2 µL of Platinum^TM^ Taq DNA Polymerase (Life Technologies Corporation, Carlsbad, CA 92008 USA) (5 U/µL) (Termofischer Scientific) and ultrapure autoclaved water to a volume of 25 µL per reaction. The PCR thermal cycling consisted of an initial incubation at 94 °C for 3 min, followed by 35 cycles (94 °C for 30 s, 56 °C for 30 s, 72 °C for 50 s) and a final extension at 72 °C for 5 min. For the second rounds: 2 µL of template derived from the first reactions, 2.5 µL of 10× PCR Buffer (KCL 50 mM; Tris HCl 10 mM; pH 9.0) (Life Technologies Corporation, Carlsbad, CA 92008 USA), 1.0 µL of MgCL_2_ (50 mM), 0.5 µL of mixed dNTPs (10 mM), 2.5 µL of each primer (10 µM), 0.2 µL of Platinum^TM^ Taq DNA ) (Life Technologies Corporation, Carlsbad, CA 92008 USA) (5 U/µL) and ultrapure autoclaved water to a volume of 25 µL per reaction. The thermal cycling was the same used in the first round.

DNA of *Sarcocystis neurona*, *Neospora caninum* and *Hammondia hammondi* was used as positive controls and ultrapure DNAse-free water as the negative control for all reactions.

PCR products were resolved on 2.0% agarose gels and viewed through UV transillumination. Amplicons of the expected sizes were treated with ExoSAP-IT (Affimetryx/Thermo Fisher Scientific, Santa Clara, CA, USA), prepared for sequencing using the Big Dye Terminator cycle sequencing kit (Applied Biosystems, Foster City, CA, USA) and sequenced in an ABI automated sequencer (ABI 3500 Genetic Analyzer, Applied Biosystems). Sequencing was performed using the same primers as in the nPCR consensus. Sequence edition and contig assemblies were done by using the software Codoncode aligner, Codoncode Corporation. Final sequences were compared with homologous available in GenBank, using the BLASTn algorithm (Table S1) (https://blast.ncbi.nlm.nih.gov/Blast.cgi).

For the phylogenies, sequences were aligned using the Clustal W program, as implemented in the BioEdit Sequence Alignment Editor [34]. The phylogenetic tree based on ITS1 was inferred using MEGA X [35], through the maximum likelihood method and T92 model of evolutionary distances. Branch supports were tested through 1000 bootstrap replications.

## 3. Results

### 3.1. Molecular Identification

**Figure 1:**
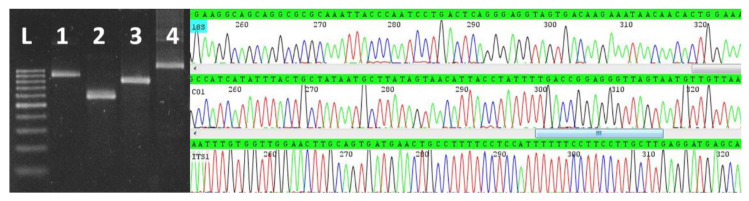
Left panel: Agarose gel electrophoresis of nPCR-ITS1 (**1**), nPCR-CO1 (**2**), and nPCR-18Sb (**3**) amplicons from *Sarcocystis* sp. ex *Stercorarius chilensis,* nPCR-ITS1 amplicons (**4**) from *Sarcocystis neurona* and Ladder Scada 100 bp, Sinapse, Inc. (**L**). Right panel: segments of the electropherogram obtained after sequencing nPCR-18Sb (**top**), nPCR-CO1 (**middle**), and nPCR-ITS1 (**bottom**) amplicons from *Sarcocystis* sp. ex *Stercorarius chilensis.*

Sixty-six tissue samples from 22 seabirds were screened by nPCR-18Sa and only two samples of pectoral muscle from two Chilean skuas were positive. None of these two samples were positive for the *T. gondii*-specific nested-PCR (nPCR-B1). The two nPCR-18Sa-positive samples were also positive by nPCR-ITS1, nPCR-18Sb and nPCR-CO1 (Figure 1, left panel). After sequencing nPCR-ITS1, nPCR-18Sb and nPCR-CO1 amplicons and removal of primer-derived sequences, 861, 783 and 547 base pairs were obtained, respectively. Fragments of the sequences obtained are shown in Figure 1, right panel. The homologous sequences of the two samples were identical to each other, thus only one set were submitted to the GenBank, under the accession numbers MW160469, MW161469, MW157378. Through BLAST searches, ITS1, CO1 and 18S genetic sequences were compared with sequences producing the most significant alignments, with query coverage ≥ 99% and percentage similarities ≥ 99.00% in the cases of CO1 and 18S. All ITS1 sequences with query cover ≥ 96% were used for analyses of the genetic sequence of the skuas.

The ITS1 fragment from the skuas showed 96.14–96.28% identity to sequences of *Sarcocystis halieti* from herring gulls (*Larus argentatus*) (MN450340–MN450356), great cormorants (*Phalacrocorax carbo*) (MH130209, JQ733513) and white-tailed sea-eagles (*Haliaeetus albicilla*) (MF946589–MF946596). *Sarcocystis* sp. from Cooper’s hawk (*Accipiter cooperii*) (KY348755), *Sarcocystis columbae* from common woodpigeons (*Columba palumbus*) (GU253885, HM125052) and herring gulls (*Larus argentatus*) (MN450338–MN450339) and *Sarcocystis corvusi* from Eurasian jackdaws (*Corvus monedula*) (JN256119) showed less than 94% sequence identity with homologous sequences from the Chilean skuas at ITS1 locus.

In contrast to ITS1, much less genetic variability was observed within the CO1 and 18S coding genes. The haplotype obtained from the skuas was named *Sarcocystis* sp. ex *Stercorarius chilensis*.

The CO1 sequences from *Sarcocystis* sp. ex *Stercorarius chilensis* were 100% identical with homologous sequences of *S. corvusi* (MH138314), *S. columbae* (MH138312) and *S. halieti* (MH138308-09; MF946583). *Sarcocystis fulicae* (MH138316), *Sarcocystis wobeseri* (MH138315), *Sarcocystis cornixi* (MH138313) and *Sarcocystis* sp. ex *Accipiter cooperii* (KY348756) differed by only one nucleotide substitution from *Sarcocystis* sp. ex *Stercorarius chilensis* at this locus. Regarding the 18S rRNA gene, the maximum percentage identity was found between *Sarcocystis* sp. ex *Stercorarius chilensis* and *S. halieti* (99.74%).

### 3.2. Phylogeny

**Figure 2:**
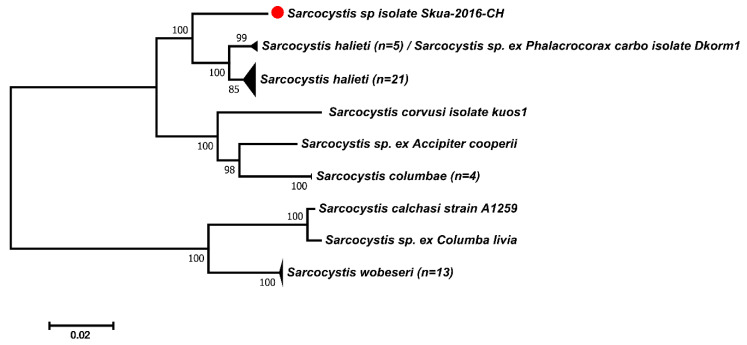
Phylogenetic tree of *Sarcocystis* species based on ITS1 sequences. The tree was constructed using the maximum likelihood method and Tamura 3 parameters nucleotide substitution model. The final alignment contained 49 sequences and 814 aligned nucleotide positions. All positions containing gaps and missing data were eliminated (complete deletion option). Numbers in branches represent bootstrap values after 1000 replicates. The red dot identifies the sequence of *Sarcocystis* sp. ex *Stercorarius chilensis*.

The ITS1-based phylogeny demonstrated that the species that shares the most recent ancestral commonality with *Sarcocystis* sp. ex *Stercorarius chilensis* was *S. halieti* (Figure 2).

These sequences were separated with high support from a major clade comprising the species *Sarcocystis* sp. ex *Columba livia* (FJ232948), *Sarcocystis calchasi* (KC733715–KC733718) and *Sarcocystis wobeseri* (MN450365–MN450373, HM159421, JN256121), which exploit Anseriformes, Charadriiformes, Columbiformes, Psittaciformes and other birds as intermediate hosts.

Altogether, the *Sarcocystis* species that were most similar to *Sarcocystis* sp. ex *Stercorarius chilensis* used birds as intermediate hosts.

## 4. Discussion

*Toxoplasma gondii* has high genotypic diversity and several new genotypes have been described in wildlife [36,37,38], which has aided to understand the shape of molecular evolution and the epidemiology of the infection [38]. Similarly, several species descriptions have been made for the genus *Sarcocystis,* most of them with the aid of molecular methods [39,40,41,42,43]. This study presents the results of a molecular screening of Sarcocystidae focusing on animal species and geographical areas where these parasites have rarely or not yet been identified. DNA of organisms of the genus *Sarcocystis* was identified in two Chilean skuas, whereas DNA of *T. gondii* were not found in any sample. The *Sarcocystis* haplotype detected in skuas was named *Sarcocystis* sp. ex *Stercorarius chilensis.*

Although all the samples tested negative for the presence of *T. gondii*, antibodies against this parasite were detected previously in 57 (43.18%) of the 132 serum samples from free-living Magellanic penguins from the same region, with titers that ranged from 20 to 320 [7]. Herein, infected animals were not encountered possibly because the sampling was insufficient to find at least one positive animal, as the prevalence of the infection in seabirds in the sampled area are not known and the sampling might have not been representative of the population surveyed. In addition, the mass of tissue that was tested might have been insufficient because of the very sparse and focal distribution of *T. gondii* cysts in the tissues of the HI, thus, digestion of samples previously to the DNA extraction and subsequent DNA detection would be more appropriate than direct DNA extraction, as used in this study [44].

Oocysts of *T. gondii* can sporulate and survive in seawater for months [45,46]. Marine mammals in different groups (cetaceans, pinnipeds and sirenians) and seabirds might become infected through consumption of water containing the oocysts. Thus, *T. gondii* oocysts from felidae feces might enter the marine environment and contaminate both the water and several invertebrate species, which could act as vectors of infection for mammals and seabirds [47]. Mice can be experimentally infected when fed with *T. gondii*-contaminated oysters (*Crassostrea virginica*) [45] proving that *T. gondii* was able to survive for several months in these mollusks [48]. Anchovies and Pacific sardines can be experimentally contaminated with *T. gondii* oocysts, which indicates that migratory filter feeders may serve as biotic vectors for this parasite [49]. Another study proved that freshwater crustaceans were able to bioaccumulate *T. gondii* oocysts. It should be noted that crustaceans are part of penguins’ and many seabirds’ food chain [50,51]. Thus, although the birds screened here were found not infected by *T. gondii*, marine fauna are at risk of acquiring the infection, by ingesting oocysts carried by transport hosts (oysters, fish and other) or through predation of intermediate hosts in the marine or in the coastal environment.

Based on molecular data, *Sarcocystis* sp. ex *Stercorarius chilensis* is an undescribed *Sarcocystis* species, closely related to *S. halieti*. The molecular identification based on ITS1, CO1 and 18S rRNA gene sequences showed a closed relationship between *Sarcocystis* sp. from Chilean skuas and other *Sarcocystis* spp. that use birds as intermediate hosts and predatory birds as definitive hosts. As expected, the most variable locus was ITS1, and phylogenies based on 18S rRNA and CO1 genes showed insufficient discrimination power to differentiate between species within the genus [39,41].

The most similar sequences to ITS1 of *Sarcocystis* sp. ex *Stercorarius chilensis* are those from *Sarcocystis* spp. that use hawks as definitive hosts. *Sarcocystis* sp. ex *Stercorarius chilensis* grouped together with *S. halieti*, a species that uses white-tailed sea-eagles (*Haliaeetus albicilla*) and Eurasian sparrowhawks (*Accipiter nisus*) as definitive hosts [39]. Other taxa found through ITS1-based BLAST searches encompass *Sarcocystis* spp. that also use hawks as definitive hosts (*Accipiter cooperii*, *Accipiter nisus*), except for *S. corvusi*, for which this information remains unknown [52,53]. Accipiter hawks (*Accipiter gentilis*, *Accipiter nisus*) are definitive hosts for *S. calchasi* [54,55,56].

Several studies have expanded the knowledge on the host specificity of *Sarcocystis*, as unequivocal identification of the parasite can be achieved after identifying sarcocysts and oocysts to species level using molecular methods. *Sarcocystis halieti* and *Sarcocystis lari* were found to have formed oocysts in the intestine of white-tailed sea eagle (*Haliaeetus albicilla*), showing for the first time the potential role of sea eagle as definitive host of those species of *Sarcocystis* [53]. Likewise, european seabirds were found to harbor several species of *Sarcocystis* after DNA of *Sarcocystis lari*, *S. wobeseri*, *S. columbae* and *S. halieti* were detected in sarcocysts infecting muscle of herring gulls (*Larus argentatus*), great black-backed gulls (*Larus marinus*) and great cormorants (*Phalacrocorax carbo*) in Lithuania [40,41,42].

The four morphologically indistinguishable *Sarcocystis* species, *Sarcocystis lari*, *S. wobeseri*, *S. columbae* and *S. halieti,* could only be differentiated in *L. argentatus* by means of ITS1 sequence analysis [42]. Likewise, only ITS1 clearly discriminated *Sarcocystis* sp. ex *Stercorarius chilensis* from *S. halieti*, which reinforces that molecular characterization using this marker is of paramount importance to distinguish closed related species within the genus.

It is well known that a single animal can host more than one *Sarcocystis* species [40]. Here, sarcocysts were not individually excised and subjected to molecular examination, notwithstanding, the possibility of mixed infected samples of skuas was discarded because single peaks and clean sequence throughout the chromatograms were obtained for each sequence. Thus, a haplotype could be confidently assigned to the samples.

Although screening *Sarcocystis* by using molecular methods without morphological characterization of parasitic structures is obviously not enough to name a new species, this procedure may provide subsides to future studies on the epidemiology of the infection and its impact on the health of marine fauna. To our knowledge, *Sarcocystis* in south American seabirds were identified only once [43], which suggests that a wide field of research on diversity of sarcocystidae can be explored on this continent.

## 5. Conclusions

Although few animals have been screened in this study and morphological characterization of the parasites was not carried out, evidence of an unprecedented haplotype of *Sarcocystis* was found in skuas from Chile, which demonstrate that molecular screening of *Sarcocystis* can be a valuable tool to prospect for new species, contributing to knowledge on the epidemiology of sarcocystosis and life cycle of *Sarcocystis*. Sporocysts shed with feces, sarcocysts in tissues or rapid dividing structures in acute sarcocystosis (schyzonts and merozoites) can be more easily and accurately identified as data on *Sarcocystis* genetic sequences increases. Nevertheless, a complete study encompassing aspects of life cycle and morphological data is necessary to fully describe *Sarcocystis* sp. ex *Stercorarius chilensis* and additional studies are needed to better understand the epidemiology of the infection and its impact on the health of marine fauna.

## Figures and Tables

**Table 1:** Primers for the detection of Sarcocystidae in tissues of seabirds from Magdalena Island, Chile.

PCR	Primers	Sequences	PCR Step ^a^	Reference
nPCR-18S	Tg18s48F	CCATGCATGTCTAAGTATAAGC	1	[28]
	Tg18s359R	GTTACCCGTCACTGCCAC	1	[28]
	Tg18s58F	CTAAGTATAAGCTTTTATACGGC	2	[28]
	Tg18s348R	TGCCACGGTAGTCCAATAC	2	[28]
nPCR-B1	T1	AGCGTCTCTCTTCAAGCAGCGTA	1	[29]
	T2	TCCGCAGCGACTTCTATCTCTGT	1	[29]
	T3	TGGGAATGAAAGAGACGCTAATGTG	2	[29]
	T4	TTAAAGCGTTCGTGGTCAACTATCG	2	[29]
nPCR-18Sb	18S9L	GGATAACCTGGTAATTCTATG	1 + 2	[32]
	18S1H	GGCAAATGCTTTCGCAGTAG	1 + 2	[32]
nPCR-CO1	COX1-227F25	GTTTTGGTAACTACTTTGTACCGAT	1	[31]
	COX1-885R25	GAAATATGCACGAGTATCTACCTCT	1	[31]
	COX1-275F22	TGTACCCACGAATTAATGCAGT	2	[31]
	COX1-844R21	GTGTGCCCATACTAGAGAACC	2	[31]
nPCR-ITS1	JS4	CGAAATGGGAAGTTTGAAC	1	[33]
	CT2c	CTGCAATTCACATTCGC	1	[30]
	JS4b	AGTCGTAACAAGGTTTCCGTAGG	2	[30]
	CT2b	TTGCGCGAGCCAAGACATC	2	[30]

^a^ Primers used in the first round of amplification (1); primers used in the second round of amplification (2); primers used in both first and second round of amplification (1 + 2).

## Data Availability

Not applicable.

## Supplementary Materials

The following are available online at https://www.mdpi.com/2076-2615/11/2/245/s1, Table S1. Results from BLAST search using sequences of ITS1, COX1 and 18S of *Sarcocystis* sp. ex *Stercorarius chilensis*.

## References

[1] Sam-Yellowe T.Y. (1996). Rhoptry organelles of the apicomplexa: Their role in host cell invasion and intracellular survival. Parasitol. Today.

[2] Morrison D.A., Bornstein S., Thebo P., Wernery U., Kinne J., Mattsson J.G. (2004). The current status of the small subunit rRNA phylogeny of the coccidia (Sporozoa). Int. J. Parasitol..

[3] Mugridge N.B., Morrison D.A., Johnson A.M., Luton K., Dubey J.P., Votýpka J., Tenter A.M. (1999). Phylogenetic relationships of the genus *Frenkelia*: A review of its history and new knowledge gained from comparison of large subunit ribosomal ribonucleic acid gene sequences. Int. J. Parasitol..

[4] Samarasinghe B., Johnson J., Ryan U. (2008). Phylogenetic analysis of Cystoisospora species at the rRNA ITS1 locus and development of a PCR-RFLP assay. Exp. Parasitol..

[5] Tenter A.M., Barta J.R., Beveridge I., Duszynski D.W., Mehlhorn H., Morrison D.A., Andrew Thompson R.C., Conrad P.A. (2002). The conceptual basis for a new classification of the coccidian. Int. J. Parasitol..

[6] Dubey J.P. (2010). Toxoplasmosis of Animals and Humans.

[7] Acosta I.C.L., Souza-Filho A.F., Muñoz-Leal S., Soares H.S., Heinemann M.B., Moreno L., González-Acuña D., Gennari S.M. (2019). Evaluation of antibodies against *Toxoplasma gondii* and *Leptospira* spp. in Magellanic penguins (*Spheniscus magellanicus*) on Magdalena Island, Chile. Vet. Parasitol. Reg. Stud. Rep..

[8] Dubey J.P.J., Calero-Bernal R., Rosenthal B.M.B., Speer C.A.C., Fayer R. (2015). Sarcocystosis of Animals and Humans.

[9] Rioseco H., Cubillos V., González H., Díaz L. (1976). Sarcosporidiosis en pudues (*Pudu pudu,* Molina, 1782) Primera comunicación en Chile. Arch. Med. Vet..

[10] Gorman T.R., Alcaíno H.A., Muñuz H., Cunazza C. (1984). *Sarcocystis* sp. in guanaco (*Lama guanicoe*) and effect of temperature on its viability. Vet. Parasitol..

[11] Sepúlveda M.A., Seguel M., Alvarado-Rybak M., Verdugo C., Muñoz-Zanzi C., Tamayo R. (2015). Postmortem findings in four South American sea lions (*Otaria byronia*) from an urban colony in Valdivia, Chile. J. Wildl. Dis..

[12] Tavares D.C., Moura J.F., de Amorim C.E., Boldrini M.A., Siciliano S. (2012). Aves, Stercorariidae, Chilean Skua *Stercorarius chilensis* Bonaparte, 1857: First documented record for the state of Espírito Santo, southeastern Brazil. Check List.

[13] Furness R.W. (1987). The Skuas.

[14] Robinson I. (2009). Seabirds. Handbook of Avian Medicine.

[15] BirdLife International Species Factsheet: Catharacta Chilensis. http://www.birdlife.org.

[16] Ludynia K., Garthe S., Luna-Jorquera G. (2005). Seasonal and regional variation in the diet of the Kelp Gull in northern Chile. Waterbirds.

[17] Yorio P., Branco J.O., Lenzi J., Luna-Jorquera G., Zavalaga C. (2016). Distribution and Trends in Kelp Gull (*Larus dominicanus*) Coastal Breeding Populations in South America. Waterbirds.

[18] Boersma P.D., Frere E., Kane O., Pozzi L.M., Pütz K., Rey A.R., Borboroglu P.G., Boersma P.D. (2013). Magellanic penguins. Penguins: Natural History and Conservation.

[19] Frere E., Gandini P., Lichtschein V. (1996). Variacion latitudinal en la dieta del pinguino de Magallanes (*Spheniscus magellanicus*) en la Costa Patagonica, Argentina. Ornitol. Neotrop..

[20] Odening K. (1998). The present state of species-systematics in *Sarcocystis* Lankester, 1882 (Protista, Sporozoa, Coccidia). Syst. Parasitol..

[21] Pan J., Ma C., Huang Z., Ye Y., Zeng H., Deng S., Hu J., Tao J. (2020). Morphological and molecular characterization of *Sarcocystis wenzeli* in chickens (*Gallus gallus*) in China. Res. Sq..

[22] Konradt G., Bianchi M.V., Leite-Filho R.V., da Silva B.Z., Soares R.M., Pavarini S.P., Driemeier D. (2017). Necrotizing meningoencephalitis caused by *Sarcocystis falcatula* in bare-faced ibis (*Phimosus infuscatus*). Parasitol. Res..

[23] Olias P., Maier K., Wuenschmann A., Reed L., Armién A.G., Shaw D.P., Gruber A.D., Lierz M. (2014). *Sarcocystis calchasi* has an expanded host range and induces neurological disease in cockatiels (*Nymphicus hollandicus*) and North American rock pigeons (*Columbia livia* f. dom.). Vet. Parasitol..

[24] Wilson T.M., Sousa S.K.H., Paludo G.R., de Melo C.B., Llano H.A.B., Soares R.M., Castro M.B. (2020). An undescribed species of *Sarcocystis* associated with necrotizing meningoencephalitis in naturally infected backyard chickens in the Midwest of Brazil. Parasitol. Int..

[25] Roeder A.D., Ritchie P.A., Lambert D.M. (2002). New DNA markers for penguins. Conserv. Genet..

[26] Pons J.M., Hassanin A., Crochet P.A. (2005). Phylogenetic relationships within the Laridae (Charadriiformes, Aves) inferred from mitochondrial markers. Mol. Phylogenet. Evol..

[27] Han Y.D., Baek Y.S., Kim J.H., Choi H.G., Kim S. (2016). Complete mitochondrial genome of the South Polar Skua *Stercorarius maccormicki* (Charadriiformes, Stercorariidae) in Antarctica. Mitochondrial DNA.

[28] Su C., Shwab E.K., Zhou P., Zhu X.Q., Dubey J.P. (2010). Moving towards an integrated approach to molecular detection and identification of *Toxoplasma gondii*. Parasitology.

[29] Yai L.E.O., Cañon-Franco W.A., Geraldi V.C., Summa M.E.L., Camargo M.C.G.O., Dubey J.P., Gennari S.M. (2003). Seroprevalence of *Neospora caninum* and *Toxoplasma gondii* antibodies in the South American opossum (*Didelphis marsupialis*) from the city of São Paulo, Brazil. J. Parasitol..

[30] Soares R.M., Lopes E.G., Keid L.B., Sercundes M.K., Martins J., Richtzenhain L.J. (2011). Identification of *Hammondia heydorni* oocysts by a heminested-PCR (hnPCR-AP10) based on the *H. heydorni* RAPD fragment AP10. Vet. Parasitol..

[31] Gondim L.F.P., Soares R.M., Tavares A.S., Silva W.B., de Jesus R.F., Llano H.A.B., Gondim L.Q. (2019). *Sarcocystis falcatula*-like derived from opossum in Northeastern Brazil: In vitro propagation in avian cells, molecular characterization and bioassay in birds. Int. J. Parasitol. Parasites Wildl..

[32] Li Z.Q., Yang Y.X., Zuo S.W., Attwood X.W., Chen Y.P., Zhang A. (2002). PCR-based RFLP analysis of *Sarcocystis cruzi* (Protozoa: Sarcocystidae) in Yunnan Province, PR China, reveals the water buffalo (*Bubalus bubalis*) as a natural intermediate host. J. Parasitol..

[33] Šlapeta J.R., Koudela B., Votýpka J., Modrý D., Hořejš R., Lukeš J. (2002). Coprodiagnosis of Hammondia heydorni in dogs by PCR based amplification of ITS 1 rRNA: Differentiation from morphologically indistinguishable oocysts of *Neospora caninum*. Vet. J..

[34] Thompson J.D., Gibson T.J., Plewniak F., Jeanmougin F., Higgins D.G. (1997). The CLUSTAL_X windows interface: Flexible strategies for multiple sequence alignment aided by quality analysis tools. Nucleic Acids Res..

[35] Kumar S., Stecher G., Li M., Knyaz C., Tamura K. (2018). MEGA X: Molecular evolutionary genetics analysis across computing platforms. Mol. Biol. Evol..

[36] Sibley L.D., Khan A., Ajioka J.W., Rosenthal B.M. (2009). Genetic diversity of *Toxoplasma gondii* in animals and humans. Philos. Trans. R. Soc. Lond. B Biol. Sci..

[37] Vitaliano S.N., Soares H.S., Minervino A.H.H., Santos A.L.Q., Werther K., Marvulo M.F.V., Siqueira D.B., Pena H.F.J., Soares R.M., Su C. (2014). Genetic characterization of *Toxoplasma gondii* from Brazilian wildlife revealed abundant new genotypes. Int. J. Parasitol. Parasites Wildl..

[38] Aguirre A.A., Longcore T., Barbieri M., Dabritz H., Hill D., Klein P.N., Lepczyk C., Lilly E.L., Milcarsky R.M.J., Su C. (2019). The one health approach to toxoplasmosis: Epidemiology, control, and prevention strategies. EcoHealth.

[39] Gjerde B., Vikøren T., Hamnes I.S. (2018). Molecular identification of *Sarcocystis halieti* n. sp., *Sarcocystis lari* and *Sarcocystis truncata* in the intestine of a white-tailed sea eagle (*Haliaeetus albicilla*) in Norway. Int. J. Parasitol. Parasites Wildl..

[40] Prakas P., Butkauskas D., Juozaitytė-Ngugu E. (2020). Molecular identification of four *Sarcocystis* species in the herring gull, *Larus argentatus*, from Lithuania. Parasit Vectors.

[41] Prakas P., Butkauskas D., Švažas S., Stanevičius V. (2018). Morphological and genetic characterisation of *Sarcocystis halieti* from the great cormorant (*Phalacrocorax carbo*). Parasitol. Res..

[42] Prakas P., Kutkiene L., Butkauskas D., Sruoga A., Zalakevicius M. (2014). Description of *Sarcocystis lari* sp. n. (Apicomplexa: Sarcocystidae) from the great black-backed gull, *Larus marinus* (Charadriiformes: Laridae), on the basis of cyst morphology and molecular data. Folia Parasitol..

[43] Acosta I.C.L., Soares R.M., Mayorga L.F.S.P., Alves B.F., Soares H.S., Gennari S.M. (2018). Occurrence of tissue cyst forming coccidia in Magellanic penguins (*Spheniscus magellanicus)* rescued on the coast of Brazil. PLoS ONE.

[44] Dubey J.P. (1988). Long-term persistence of *Toxoplasma gondii* in tissues of pigs inoculated with *T. gondii* oocysts and effect of freezing on viability of tissue cysts in pork. Am. J. Vet. Res..

[45] Lindsay D.S., Collins M.V., Mitchell S.M., Cole R.A., Flick G.J., Wetch C.N., Lindquist A., Dubey J.P. (2003). Sporulation and survival of *Toxoplasma gondii* oocysts in seawater. J. Eukaryot. Microbiol..

[46] Fayer R., Dubey J.P., Lindsay D.S. (2004). Zoonotic protozoa: From land to sea. Trends Parasitol..

[47] Cole R.A., Lindsay D.S., Howe D.K., Roderick C.L., Dubey J.P., Thomas N.J., Baeten L.A. (2000). Biological and molecular characterization of *Toxoplasma gondii* strains obtained from southern sea otters (*Enhydra lutris nereis*). J. Parasitol..

[48] Lindsay D.S., Phelps K.K., Smith S.A., Flick G., Sumner S.S., Dubey J.P. (2001). Removal of *Toxoplasma gondii* oocysts from sea water by eastern oysters (*Crassostrea virginica*). J. Eukaryot. Microbiol..

[49] Lindsay D.S., Collins M.V., Mitchell S.M., Wetch C.N., Rosypal A.C., Flick G.J., Zajac A.M., Lindquist A., Dubey J.P. (2004). Survival of *Toxoplasma gondii* oocysts in Eastern oysters (*Crassostrea virginica*). J. Parasitol..

[50] Massie G.N., Ware M.W., Villegas E.N., Black M.W. (2010). Uptake and transmission of *Toxoplasma gondii* oocysts by migratory, filter-feeding fish. Vet. Parasitol..

[51] Bigot-Clivot A., Ladeiro M.P., Lepoutre A., Bastien F., Bonnard I., Dubey J.P., Villena I., Aubert D., Geffard O., François A. (2016). Bioaccumulation of *Toxoplasma* and *Cryptosporidium* by the freshwater crustacean *Gammarus fossarum*: Involvement in biomonitoring surveys and trophic transfer. Ecotoxicol. Environ. Saf..

[52] Mayr S.L., Maier K., Müller J., Enderlein D., Gruber A.D., Lierz M. (2016). *Accipiter hawks* (Accipitridae) confirmed as definitive hosts of *Sarcocystis turdusi**, Sarcocystis cornixi* and *Sarcocystis* sp. ex *Phalacrocorax carbo*. Parasitol. Res..

[53] Lindsay D.S., Verma S.K., Scott D., Dubey J.P., von Dohlen A.R. (2017). Isolation, molecular characterization, and in vitro schizogonic development of *Sarcocystis* sp. ex *Accipiter cooperii* from a naturally infected Cooper’s hawk (*Accipiter cooperii*). Parasitol. Int..

[54] Olias P., Olias L., Krücken J., Lierz M., Gruber A.D. (2011). High prevalence of *Sarcocystis calchasi* sporocysts in European *Accipiter hawks*. Vet. Parasitol..

[55] Olias P., Gruber A.D., Hafez H.M., Heydorn A.O., Mehlhorn H., Lierz M. (2010). *Sarcocystis calchasi* sp. nov. of the domestic pigeon (*Columba livia* f. domestica) and the Northern goshawk (*Accipiter gentilis*): Light and electron microscopical characteristics. Parasitol. Res..

[56] Olias P., Olias L., Lierz M., Mehlhorn H., Gruber A.D. (2010). *Sarcocystis calchasi* is distinct to *Sarcocystis columbae* sp. nov. from the wood pigeon (*Columba palumbus*) and Sarcocystis sp. from the sparrow hawk (*Accipiter nisus*). Vet. Parasitol..

