# Supplementary material for: Molecular Characterization of New Haplotype of Genus Sarcocystis in Seabirds from Magdalena Island, Southern Chile
Source: Animals (Basel). 2021 Jan 20;11(2):245. doi: 10.3390/ani11020245 (PMC7909292; doi:10.3390/ani11020245)
Supplement: Supplementary file 1 [file animals-11-00245-s001.pdf]

Table S1. Results from BLAST search using sequences of ITS1, COX1 and 18S of *Sarcocystis* sp. ex *Stercorarius chilensis*

Locus	Genbank number	Species	Percent identity	N° gaps	N° of nucleotide substitutions	Sequences obtained from:	
						Definitive host	Intermediate host
18S	MH130211.1; MF946587.1	*Sarcocystis halieti*	99.74%	0	2	*Haliaeetus albicilla*	*Phalacrocorax carbo*
18S	KY348753.1	*Sarcocystis* sp. ex *Accipiter cooperii*	99.74%	0	2	*Accipiter cooperii*	
18S	JQ733511.1	*Sarcocystis* sp. ex *Phalacrocorax carbo* is. Dkom1	99.74%	0	2	Unknown	*Phalacrocorax carbo*
18S	GQ245670.1	*Sarcocystis* sp. ex *Columba livia*	99.74%	0	2	Unknown	*Columba livia*
18S	EU502869.2	*Sarcocystis* sp. ex *Anser albifrons* is. 09	99.74%	0	2	Unknown	*Anser albifrons*
18S	EU810398.1	*Sarcocystis* sp. CH1918-03	99.74%	0	2	*Accipiter cooperii*	
18S	MG273671.1	*Sarcocystis fulicae*	99.62%	0	3	Unknown	*Fulica atra*
18S	JN256117.1	*Sarcocystis corvusi*	99.62%	0	3	Unknown	*Corvus monedula*
18S	GU187943.1	*Sarcocystis* sp. MK-2010	99.62%	0	3	Unknown	*Nyctereutes procyonoides viverrinus*
18S	HM125054.1; GU253883.1	*Sarcocystis columbae*	99.62%	0	3		*Columba palumbus*
18S	EU810399.1	*Sarcocystis* sp. CH327-04	99.62%	0	3	*Accipiter cooperii*	Unknown
18S	EU810397.1	*Sarcocystis* sp. CH255-04	99.62%	0	3	*Accipiter cooperii*	Unknown
18S	KY994649.1	*Sarcocystis jamaicensis*	99.49%	0	4	*Buteo jamaicensis*	Unknown
18S	EU810402.1	*Sarcocystis* sp. CH13-04	99.49%	0	4	*Accipiter cooperii*	Unknown
18S	KM362428.1	Sarcocystis sp. 1 SN-2013	99.49%	0	4	Unknown	*Canis familiaris*
18S	JF975681.1	*Sarcocystis turdusi*	99.49%	0	4	Unknown	*Turdus merula*
18S	GU253884.1	*Sarcocystis* sp. ex *Accipiter nisus*	99.49%	0	4	*Accipiter nisus*	Unknown
18S	EU810401.1	*Sarcocystis* sp. CH318-04	99.49%	0	4	*Accipiter cooperii*	Unknown
18S	EU810395.1	*Sarcocystis* sp. RTH378-04	99.49%	0	4	*Buteo jamaicensis*	Unknown
18S	MF946588.1; JQ733508.1	*Sarcocystis lari*	99.36%	0	5	*Haliaeetus albicilla*	*Larus marinus*
18S	JN256677.1	*Sarcocystis* sp. CO1	99.36%	0	5	Unknown	*Canis familiaris*

18S	EU810396.1	*Sarcocystis* sp. RH168-04	99.36%	0	5	*Buteo lineatus*	
18S	MT036251.1; MT036248.1	*Sarcocystis lutrae*	99.23%	1	6	Unknown	*Nyctereutes procyonoides*
18S	MN169125.1	*Sarcocystis neurona*	99.23%	0	6	Unknown	*Felis domesticus*
18S	MF596215.1.-MF596216.1; MG372102.1-MG372103.1; MG272286.1-MG272295.1	*Sarcocystis lutrae*	99.23%	1	6		*Not especified Lutra luta, Meles meles Mustelidae*
18S	KX470746.1	*Sarcocystis* sp. isolate 5	99.23%	0	6	*Didelphis aurita*	Unknown
18S	KT207459.1	*Sarcocystis speeri*	99.23%	0	6	*Didelphis albiventris*	Unknown
18S	KM657769.1	*Sarcocystis lutrae*	99.23%	1	6	Unknown	*Lutra lutra*
18S	EU810400.1	Sarcocystis sp. CH47-04	99.23%	0	6	*Accipiter cooperii*	
18S	KP681854.1	*Sarcocystis speeri*	99.11%	0	7	Unknown	
18S	MK803217.1	*Sarcocystis* sp. isolate Sarco-BA1	99.11%	0	7	*Didelphis spp.*	
18S	MH626537.1	*Sarcosytis falcatula*	99.11%	0	7		*Trichoglossus moluccanus*
18S	KX610767.1-KX610769.1	Sarcocystis sp. isolate B200	99.11%	0	7	*Didelphis spp.*	
18S	AF009245.1	*Frenkelia glareoli*	99.11%	0	7	Unknown	*Clethrionomys glareolus*
18S	KJ957839.1	*Sarcocystis neurona*	99.11%	0	7	Unknown	*Canis familiaris*
18S	EU553478.2	*Sarcocystis cornixi*	98.98%	2	8	Unknown	*Corvus cornix*
COX1	MH138314.1	*Sarcocystis corvusi*	100.00%	0	0	Unknown	
COX1	MH138312.1	*Sarcocystis columbae*	100.00%	0	0	Unknown	
COX1	MH138308.1-MH138309.1; MF946583.1	*Sarcocystis halieti*	100.00%	0	0	*Haliaeetus albicilla*	*Phalacrocorax carbo*
COX1	MH138316.1	*Sarcocystis fulicae*	99.82%	0	1	Unknown	*Fulica atra*
COX1	MH138315.1	*Sarcocystis wobeseri*	99.82%	0	1	Unknown	
COX1	MH138313.1	*Sarcocystis cornixi*	99.82%	0	1		*Corvus cornix*
COX1	KY348756.1	*Sarcocystis* sp. ex *Accipiter cooperii*	99.82%	0	1	*Accipiter cooperii*	
COX1	MT036254.1; MT036250.1; MG273661.1-MG273670.1; MF596284.1-MF596285; MG372106.1-MG372107.1;	*Sarcocystis lutrae*	99.63%	0	2	Unknown	*Nyctereutes procyonoides, Procyon lotor Neovison vison, Martes foina, Meles meles, Lutra*

	KF601326.1						*lutra, Mustela putorius, Vulpes lagopus*
COX1	MF596283.1-MF946584.1	*Sarcocystis lari*	99.63%	0	2	Unknown	*Larus marinus*
COX1	KT588511.1-KT588518.1	*Sarcocystis turdusi*	99.63%	0	2	Unknown	*Turdidae, Muscicapidae*
COX1	KY994652.1	*Sarcocystis jamaicensis*	99.45%	0	3	*Buteo jamaicensis*	Unknown
COX1	MF162317.1	*Sarcocystis* sp. SKV-2017a	99.09%	0	5	*Strix varia*	Unknown
COX1	MH469240.1	*Sarcocystis caninum*	98.72%	0	7	Unknown	*Canis familiaris*
ITS1	MN450340.1-MF450341.1; MH130209.1	*Sarcocystis halieti*	96.22%	0	31	Unknown	*Larus argentatus* *Phalacrocorax carbo*
ITS1	JQ733513.1	*Sarcocystis* sp. ex *Phalacrocorax carbo* isolate Dkorm1	96.22%	1	31	Unknown	*Phalacrocorax carbo*
ITS1	MN450342.1-MN450356.1; MF946589.1	*Sarcocystis halieti*	96.10%	0	32	*Haliaeetus albicilla*	*Larus argentatus*
ITS1	MF946590.1; MF946593.1	*Sarcocystis halieti*	95.98%	0	33	*Haliaeetus albicilla*	Unknown
ITS1	MF946596.1	*Sarcocystis halieti*	95.85%	2	34	*Haliaeetus albicilla*	Unknown
ITS1	MF946591.1-MF946592.1	*Sarcocystis halieti*	95.73%	0	35	*Haliaeetus albicilla*	Unknown
ITS1	MF946594.1-MF946595.1	*Sarcocystis halieti*	95.61%	0	36	*Haliaeetus albicilla*	Unknown
ITS1	KY348755.1	*Sarcocystis sp. ex Accipiter cooperii*	92.95%	4	58	*Accipiter cooperii*	Unknown
ITS1	MN450338.1-MN450339.1; GU253885.1	*Sarcocystis columbae*	91.99%	5	66	Unknown	*Larus argentatus* *Columba palumbus*
ITS1	HM125052.1	*Sarcocystis columbae*	91.87%	6	67	Unknown	*Columba palumbus*
ITS1	JN256119.1	*Sarcocystis corvusi*	91.06%	8	74	Unknown	*Corvus monedula*
ITS1	FJ232948.1	*Sarcocystis sp. ex Columba livia*	83.89%	27	134	Unknown	*Columba livia*
ITS1	KC733715.1-KC733718.1	*Sarcocystis calchasi*	83.69%	29	136	Unknown	Psittacidae
ITS1	MN450370.1-MN450373.1; HM159421.1	*Sarcocystis wobeseri*	83.55%	35	138	Unknown	*Larus argentatus*
ITS1	MN450365.1-MN450369.1; GU475111.1	*Sarcocystis wobeseri*	83.43%	35	139	Unknown	*Larus argentatus* *Anas platyrhynchos, Branta leucopsis*

ITS1	JN256121.1	*Sarcocystis wobeseri*	83.31%	35	140	Unknown	*Anas platyrhynchos*
